# Supplementary material for: Empirical methods for controlling false positives and estimating confidence in ChIP-Seq peaks
Source: BMC Bioinformatics. 2008 Dec 5;9:523. doi: 10.1186/1471-2105-9-523 (PMC2628906; doi:10.1186/1471-2105-9-523)
Supplement: Additional File 1 — A variety of html documents from the USeq web site detailing the available applications, their best usage, output file type descriptions, command line menus, etc. [file 1471-2105-9-523-S1.zip › USeqUserGuides/JavaDoc.zip!/JavaDoc/trans/main/ScanChromosomesCNV.html]

ScanChromosomesCNV


|  |  |  |
| --- | --- | --- |
| **Overview** \| **Package** \| **Class** \| **Use** \| **Tree** \| **Deprecated** \| **Index** \| **Help** | |  |
| **PREV CLASS**   **NEXT CLASS** | **FRAMES**    **NO FRAMES**     **All Classes** |  |
| SUMMARY: NESTED \| FIELD \| CONSTR \| METHOD | DETAIL: FIELD \| CONSTR \| METHOD |  |


---


## trans.main Class ScanChromosomesCNV

```
java.lang.Object
  trans.main.ScanChromosomesCNV
```

---

``` public class ScanChromosomesCNV extends java.lang.Object ```

Scores windows of intensity values using several tests.

---

**Constructor Summary**

`ScanChromosomesCNV(java.lang.String[] args)`


| **Method Summary** | |
| --- | --- |
| `void` | `addPermutatedScoresToHistogram(java.util.ArrayList windowsAL)`             Uses random permutation of the cel file labels to estimate a null distribution, store random scores in a histogram for p-value and FDR estimations. |
| `void` | `buildExtensionSetThreshold()` |
| `void` | `calculateRandomPermutationConfidenceScores()`             Takes the histogram of random permutation scores and calculates a p-value and FDR for each real score. |
| `static boolean` | `checkSizes(float[][] replicasValues)`             Checks to see if the number of values are the same given float[replica#][values]. |
| `void` | `convertRatiosToPValues()` |
| `static float[][]` | `fetchIntensities(java.io.File[] directories, java.lang.String fileName, boolean randomizeData)`             Fetches a float[] from each of the directories given a fileName. |
| `float[][]` | `getControlIntensities()` |
| `int` | `getNumPermutations()` |
| `int[]` | `getPositions()` |
| `java.io.File` | `getResultsDirectory()` |
| `static java.lang.String[]` | `getSCORE_DESCRIPTION()` |
| `float[][]` | `getTreatmentIntensities()` |
| `Window[]` | `getWindows()` |
| `void` | `importScores(Window[] windows, java.io.File importFile)`             Reads in a file of scores from a qvalue output file Also makes gr files for the qValues. |
| `static void` | `main(java.lang.String[] args)` |
| `void` | `makeBarFiles(Window[] windows, java.io.File directory, int scoreIndex, boolean stairStep)`             Makes xxx.bar.zip files from a Window[], one per chromosome. |
| `void` | `makeChromosomeWideScores()`             Makes chromosome wide scores. |
| `void` | `makeDirectories()`             Makes directories and files to hold point gr files and tempRatios |
| `static void` | `printDocs()` |
| `void` | `printOligoRatios()`             Prints log2 oligo ratios as a chromosome specific xxx.bar file. |
| `void` | `processArgs(java.lang.String[] args)`             This method will process each argument and assign new varibles |
| `double` | `scoreWindow(int testWindowIndex)`             Scores a window using a pseudo median or trimmed mean on oligo relative differences. |
| `void` | `shuffleIntensityLabels()`             Combines and shuffles the treatment and control intensities. |
| `void` | `shuffleIntensityPositions()`             Shuffles the treatment and control intensities (seperately). |

**Methods inherited from class java.lang.Object**

`equals, getClass, hashCode, notify, notifyAll, toString, wait, wait, wait`

**Constructor Detail**

### ScanChromosomesCNV

```
public ScanChromosomesCNV(java.lang.String[] args)
```


**Method Detail**

### calculateRandomPermutationConfidenceScores

```
public void calculateRandomPermutationConfidenceScores()
```

:   Takes the histogram of random permutation scores and calculates a p-value and FDR for each real score.

---


### addPermutatedScoresToHistogram

```
public void addPermutatedScoresToHistogram(java.util.ArrayList windowsAL)
```

:   Uses random permutation of the cel file labels to estimate a null distribution,
    store random scores in a histogram for p-value and FDR estimations.

---


### shuffleIntensityPositions

```
public void shuffleIntensityPositions()
```

:   Shuffles the treatment and control intensities (seperately).

---


### shuffleIntensityLabels

```
public void shuffleIntensityLabels()
```

:   Combines and shuffles the treatment and control intensities.

---


### scoreWindow

```
public double scoreWindow(int testWindowIndex)
```

:   Scores a window using a pseudo median or trimmed mean on oligo relative differences.
    If the number of relative differences exceed the maxScores then they are randomly
    sampled to the number of maxScores prior to running the pseudo median.
    Returns 0 if using trm mean and window size < 3.

    :   **Returns:**: trimmed mean average or pseudo median layered relative difference score -2 to 2.

---


### makeBarFiles

```
public void makeBarFiles(Window[] windows,
                         java.io.File directory,
                         int scoreIndex,
                         boolean stairStep)
```

:   Makes xxx.bar.zip files from a Window[], one per chromosome.

---


### convertRatiosToPValues

```
public void convertRatiosToPValues()
```

---


### printOligoRatios

```
public void printOligoRatios()
```

:   Prints log2 oligo ratios as a chromosome specific xxx.bar file.
    Convert to print a bar file directly!

---


### makeDirectories

```
public void makeDirectories()
```

:   Makes directories and files to hold point gr files and tempRatios

---


### makeChromosomeWideScores

```
public void makeChromosomeWideScores()
```

:   Makes chromosome wide scores.

---


### importScores

```
public void importScores(Window[] windows,
                         java.io.File importFile)
```

:   Reads in a file of scores from a qvalue output file
    Also makes gr files for the qValues.
    Saves score in last position of the window.scores[].

---


### main

```
public static void main(java.lang.String[] args)
```

---


### processArgs

```
public void processArgs(java.lang.String[] args)
```

:   This method will process each argument and assign new varibles

---


### printDocs

```
public static void printDocs()
```

---


### getSCORE\_DESCRIPTION

```
public static java.lang.String[] getSCORE_DESCRIPTION()
```

---


### buildExtensionSetThreshold

```
public void buildExtensionSetThreshold()
```

---


### fetchIntensities

```
public static float[][] fetchIntensities(java.io.File[] directories,
                                         java.lang.String fileName,
                                         boolean randomizeData)
```

:   Fetches a float[] from each of the directories given a fileName. Returns null
    if a file cannot be read or it is not a serialized float[].

    :   **Returns:**: float[replicas][intensities]

---


### checkSizes

```
public static boolean checkSizes(float[][] replicasValues)
```

:   Checks to see if the number of values are the same given float[replica#][values].

---


### getControlIntensities

```
public float[][] getControlIntensities()
```

---


### getNumPermutations

```
public int getNumPermutations()
```

---


### getPositions

```
public int[] getPositions()
```

---


### getResultsDirectory

```
public java.io.File getResultsDirectory()
```

---


### getTreatmentIntensities

```
public float[][] getTreatmentIntensities()
```

---


### getWindows

```
public Window[] getWindows()
```


---


|  |  |  |
| --- | --- | --- |
| **Overview** \| **Package** \| **Class** \| **Use** \| **Tree** \| **Deprecated** \| **Index** \| **Help** | |  |
| **PREV CLASS**   **NEXT CLASS** | **FRAMES**    **NO FRAMES**     **All Classes** |  |
| SUMMARY: NESTED \| FIELD \| CONSTR \| METHOD | DETAIL: FIELD \| CONSTR \| METHOD |  |


---
